# MicroRNA-133a Suppresses Multiple Oncogenic Membrane Receptors and Cell Invasion in Non-Small Cell Lung Carcinoma

**DOI:** 10.1371/journal.pone.0096765

**Published:** 2014-05-09

**Authors:** Lu-Kai Wang, Tzu-Hung Hsiao, Tse-Ming Hong, Hsuan-Yu Chen, Shih-Han Kao, Wen-Lung Wang, Sung-Liang Yu, Ching-Wen Lin, Pan-Chyr Yang

**Affiliations:** 1 Graduate Institute of Life Sciences, National Defense Medical Center, Taipei, Taiwan; 2 Department of Internal Medicine, College of Medicine, National Taiwan University, Taipei, Taiwan; 3 Institute of Clinical Medicine, National Cheng Kung University, Tainan, Taiwan; 4 Institute of Statistical Science, Academia Sinica, Taipei, Taiwan; 5 Department of Clinical Laboratory Sciences and Medical Biotechnology College of Medicine, National Taiwan University, Taipei, Taiwan; 6 Institute of Biomedical Sciences, Academia Sinica, Taipei, Taiwan; 7 NTU Center of Genomic Medicine, College of Medicine, National Taiwan University, Taipei, Taiwan; Institute of Biomedical Sciences, Taiwan

## Abstract

Non-small cell lung cancers (NSCLCs) cause high mortality worldwide, and the cancer progression can be activated by several genetic events causing receptor dysregulation, including mutation or amplification. MicroRNAs are a group of small non-coding RNA molecules that function in gene silencing and have emerged as the fine-tuning regulators during cancer progression. MiR-133a is known as a key regulator in skeletal and cardiac myogenesis, and it acts as a tumor suppressor in various cancers. This study demonstrates that miR-133a expression negatively correlates with cell invasiveness in both transformed normal bronchial epithelial cells and lung cancer cell lines. The oncogenic receptors in lung cancer cells, including insulin-like growth factor 1 receptor (IGF-1R), TGF-beta receptor type-1 (TGFBR1), and epidermal growth factor receptor (EGFR), are direct targets of miR-133a. MiR-133a can inhibit cell invasiveness and cell growth through suppressing the expressions of IGF-1R, TGFBR1 and EGFR, which then influences the downstream signaling in lung cancer cell lines. The cell invasive ability is suppressed in IGF-1R- and TGFBR1-repressed cells and this phenomenon is mediated through AKT signaling in highly invasive cell lines. In addition, by using the in *vivo* animal model, we find that ectopically-expressing miR-133a dramatically suppresses the metastatic ability of lung cancer cells. Accordingly, patients with NSCLCs who have higher expression levels of miR-133a have longer survival rates compared with those who have lower miR-133a expression levels. In summary, we identified the tumor suppressor role of miR-133a in lung cancer outcome prognosis, and we demonstrated that it targets several membrane receptors, which generally produce an activating signaling network during the progression of lung cancer.

## Introduction

Lung cancer mortality, especially in non-small cell lung cancers (NSCLCs), remains the leading therapeutic issue in cancer treatment worldwide [1], [2]. Due to the diversity of histology and genetic changes in NSCLC, new drugs offer an improved overall survival only to a small proportion of the patients while the majority can only be treated with palliative chemotherapy [3]. The strategy of targeted therapy, which is based on the driver manipulation and druggable target detection that can quickly and precisely distinguish the molecular differences between each individual patient and offer personalized treatment, is the most promising advancement in the management of NSCLC patients [2].

The dysregulation of receptor signaling pathways, such as receptor tyrosine kinases (RTKs) and transforming growth factor-beta receptors (TGFBRs), has been identified as the major cause for lung cancer formation [4], [5]. The genomic amplification, mutation or rearrangement of these receptors may lead to persistent activation, causing the activation of cell survival signaling and cellular transformation in addition to increasing the invasive ability as observed in various lung tumors. In NSCLC, up to 50% in adenocarcinomas of Asian patients harbor activating mutations of EGFR [6], and approximately 20% of squamous cell carcinomas have fibroblast growth factor receptor 1 (FGFR1) amplifications [7]. IGF-1R is highly expressed in epithelial differentiated NSCLC tumors, and therefore, blocking IGF-1R signaling inhibits the proliferation and survival of cancer cells [8]. TGF-β signaling is mediated by two specific cellular serine/threonine kinase receptors, namely TGFBR1 and TGFBR2, and it can induce EMT in tumor cells in association with the acquisition of motility and invasive properties [9].

MicroRNAs (miRNAs), a group of non-coding RNAs, can repress the expression of multiple target genes through endogenous RNA interference machinery [10], [11]. MiRNAs are dysregulated in a variety of human cancers, and specific signatures of aberrantly expressed miRNAs harbor diagnostic, prognostic and therapeutic implications. Increasing evidence suggests that miRNAs confer delicate biological processes and provides robustness via regulation of target networks [12]. In malignant lung cancer, oncogenic miR-135b promotes cell invasion and metastasis by modulating LATS2, β-TrCP, NDR2 and LZTS1 in the Hippo pathway [13]. Conversely, the tumor suppressor miR-486 directly targets the components of insulin growth factor (IGF) signaling, including IGF-1, IGF-1R, and p85a, in lung cancer [14]. These studies indicate that a specific miRNA may regulate multiple targets that belong to a signaling pathway or that share similar biological functions. Therefore, miRNAs may become therapeutic targets in lung cancer treatment.

MiR-133a, which belongs to the miR-133 family, was first identified as a muscle-specific miRNA, and it plays an important role in myoblast proliferation and differentiation during embryonic muscle development. Dysregulated miR-133a results in cardiovascular diseases and skeletal muscle dysfunctions, thereby indicating that miR-133a is required for the functional maintenance of muscle cells [15]. Recently, an increasing number of reports have shown that miR-133a acts as a tumor suppressor in several cancers, including prostate cancer, bladder cancer and head and neck squamous cell carcinoma [16]–[18]. In addition, the expression level of miR-133a is significantly reduced in lung squamous cell carcinoma [19], implying that miR-133a may also mediate lung cancer progression. However, the detailed mechanism of how miR-133a regulates the development of NSCLC has not yet been thoroughly validated.

In this study, we explored the functions of miR-133a and identified multiple oncogenic receptors as its downstream targets in lung cancer. We provided evidence that miR-133a may downregulate several membrane receptors that activate and participate in cross-talk through downstream signaling using the same mediators in cancer cells. Our results also confirmed that miR-133a can suppress lung cancer invasion, metastasis and proliferation. Most importantly, the miR-133a expression level is significantly associated with NSCLC clinical outcome, implying that miR-133a has potential to be a therapeutic and prognostic target for lung cancer treatment.

## Materials and Methods

### Cell culture and antibodies

A549 (a human adenocarcinoma alveolar basal epithelial cell line) and H1299 (a human non-small cell lung carcinoma cell line derived from the lymph nodes) cells were purchased from the American Type Culture Collection (ATCC, Manassas, Virginia). EKVX and H23 lung cancer cell lines were purchased from the Developmental Therapeutics Program of the National Cancer Institute (NCI, Bethesda, Maryland). The H441 cell line was a kind gift from Dr. Win-Ping Deng (Institute of Biomedical Materials and Engineering, Taipei Medical University) [20]. The BEAS-2B cell line was a kind gift from Dr. Reen Wu (Departments of Internal Medicine, University of California at Davis) [21]. The human lung adenocarcinoma cell lines CL1-1, CL1-1, CL1-5, and CL1-5-F4 were derived from *in vitro* transwell and *in vivo* metastasis selection as previously described [22]. The BES-2B, EKVX, H23, H441 and CL series cell lines were maintained in RPMI media supplemented with 10% fetal bovine serum (FBS). The H1299 and A549 cells were maintained in DMEM with 10% FBS.

The primary antibodies used for immunoblot analysis were rabbit anti-EGFR (Santa Cruz Biotechnology, CA, USA), rabbit anti-IGF-1Rβ (Santa Cruz Biotechnology), rabbit anti-TGFBR1 (Santa Cruz Biotechnology), rabbit anti-FGFR1, rabbit anti-AKT, rabbit anti-phospho AKT (ser473) (Cell Signaling Technology, Inc., MA, USA) and mouse anti-β-actin (Santa Cruz Biotechnology) antibodies.

### Gene and miRNA expression profiles

The microRNA expression levels of lung adenocarcinoma were retrieved from the Cancer Genome Atlas (TCGA) data portal. The level 3 data of miRNA-seq was downloaded. The miR-133a expression levels of 480 samples, which contained 434 tumors and 46 normal lung tissues, were extracted. The log 10 magnitudes of reads per million (RPM) were applied to a t-test to estimate the statistical significance. To identify the miR-133a target genes, the GSE20028 and GSE26032 datasets, which contains expression profiles of miR-133a-transfected cancer cells vs. control cells, was downloaded and analyzed. The fold changes of EGFR, TGFBR1, FGFR and IGF-1R were estimated.

### Quantitative PCR analysis

Total RNA was isolated using TRIZOL reagent (Invitrogen, Carlsbad, CA) according to the standard protocol. The mature miR-133a and endogenous control RNU48 were analyzed using TaqMan MicroRNA Assays (Applied Biosystems, Foster City, CA). Briefly, 30 ng of total RNA was reverse-transcribed via SuperScript-III Reverse Transcriptase (Invitrogen, Carlsbad, CA). The cDNA was amplified with a TaqMan 2× Universal Master Mix (Applied Biosystems), and miRNA-specific real-time PCR was performed using an ABI 7500 real-time PCR system.

### Invasion assay

Transwell chambers (8-µm pore size; BD Falcon, Franklin Lakes, NJ) were coated with the appropriate amount of 30 µg Matrigel (BD Biosciences, San Jose, CA), and 2.5×10^4^ cells were suspended in 10% NuSerum-containing media (Gibco BRL, Grand Island, NY, USA), seeded in the chamber, and cultured for 20 hours (CL1-5 and A549) or 24 hours (BEAS-2B and CL1-1). To prevent the activation of AKT, 1×10^5^ cells were pre-treated with LY294002 (50 µM for the BEAS-2B cells) or AKT1/2 inhibitor (7.5 µM for the CL1-5 cells) (Sigma-Aldrich, St. Louis, MO) for 24 hours, and then cells were seeded in the chamber with LY294002- or AKT inhibitor-containing media. Cells that invaded the chamber from top to bottom were fixed with methanol and stained with a 50 µg/mL solution of propidium iodide (Sigma-Aldrich, St. Louis, MO). The propidium iodide-positive signal was quantified using the Analytical Imaging Station software package. Each sample was assayed in triplicate.

### Lentiviral vector transduction

Pre-miR-133a-encoding sequences were subcloned into the pLKO-AS2.neo vector (obtained from the National RNAi Core Facility in Academia Sinica, Taipei, Taiwan), and lentiviral vectors were prepared in accordance with standard protocols. CL1-5 and A549 cells were infected by lentiviruses with the same multiplicity of infection (MOI = 3) in medium containing polybrene (8 µg/mL). One day after infection, the cells were treated with G418 to derive a pool of neomycin-resistant clones.

### Luciferase reporter assay

The 3′UTR regions of receptors from the cDNA of CL1-0 cell line were subcloned into pGL3 basic vector [3′UTR region of receptors/number of miR-133a target sequence: EGFR (18-972/2), FGFR1 (147-625/2), IGF-1R (3852-4209/1), TGFBR1 (1981-2287/1) and INSR (691-1099/1)]. One day before transfection, CL1-0 cells were seeded in 12-well plates at a concentration of 2.5×10^4^ per well. Next, 200 or 500 ng of the pLKO-AS2.neo vector or pLKO-AS2 miR-133a plasmid was co-transfected with 50 ng of pGL3-target gene-3′UTR. The Renilla luciferase plasmid (pRL-TK, Promega, Madison, WI) was co-transfected as a transfection control. Cells were lysed 36 hours after transfection, and luciferase activity was measured using a Dual-Luciferase system (Promega, Madison, WI) according to the manufacturer's protocol.

### Human phospho-receptor tyrosine kinase array

The phosphorylation levels of receptors were detected by the Human Phospho-Receptor Tyrosine Kinase Array Kit (R&D systems Inc, Minneapolis, MN) according to manufacturer's instructions. Briefly, capture and control antibodies were spotted in duplicates on nitrocellulose membranes and incubated overnight with 300 µg of protein lysate. The membrane was then washed extensively with the provided buffer and further incubated with a pan anti-phospho-tyrosine antibody conjugated to horseradish peroxidase (HRP). After incubation, arrays were washed and visualized using enhanced chemiluminescence (ECL) (Amersham, Buckinghamshire, UK).

### Transfection and cell treatments

Transient knockdown experiments were conducted with the human siRNA-SMARTpool of IGF1R, EGFR, TGFBR1 and siControl (Thermo Scientific, Wilmington, DE) for oncogenic receptors; anti-miR inhibitor and anti-Ctl (Invitrogen, Carlsbad, CA) for miR-133a. CL1-5 or A549 cells were transfected with indicated siRNAs or anti-miRNA inhibitor by RNAiFect transfection reagent (Qiagen,Valencia, CA) according to manufacturer's protocols. The cells were used for invasion experiments 48 hours post-transfection and cell numbers were counted 72 hours post-transfection.

BEAS-2B cells were seeded in 6-well plates at a concentration of 1×10^5^ for treatment with 50 µM LY294002 (Sigma-Aldrich, St. Louis, MO). The cells were used for invasion and proliferation assays 48 and 72 hours post-treatment, respectively.

Cells were seeded in 60-mm dishes at a concentration of 2×10^5^ for treatment with rIGF-1 (R&D Systems Inc., MN, USA) or human TGF-β-1 (PeproTech, Rocky Hill, NJ). After serum starvation for 8 hours, CL1-5 and A549 cells were treated with 250 ng/ml rIGF-1 for 30 and 2.5 min, respectively. For TGF-β-1 stimulation, A549 cells were treated with 0.2 ng/ml TGF-β-1 for 5 min.

### Experimental metastasis *in vivo*

0.1 ml of PBS containing single-cell suspension of CL1-5/AS2-Neo vector (Ctl) or CL1-5/AS2-Neo-miR-133a-expressing cells (1×10^6^) was injected into the lateral tail veins of 6-week-old NOD SCID mice (LASCO). The death of tumor cell-injected mice were recorded, the remaining mice were sacrificed at 39 days, and their lungs were examined for metastasis (n = 9 per group). The lungs were removed and fixed in 10% formalin and the numbers of lung nodules were counted under a dissecting microscope.

### Ethics statement, clinical lung cancer samples and immunohistochemistry

The Institutional Review Board of the Taichung Veterans General Hospital approved this investigation, and informed written consent was obtained from all patients involved in this study. Frozen lung cancer specimens from 112 patients who underwent surgical resection of NSCLC were analyzed for the expression of miR-133a. None of the patients had received adjuvant chemotherapy. MicroRNA expression profiling was performed using a TaqMan MicroRNA Assay Kit (Applied Biosystems, Foster City, CA) and the ABI PRISM 7900 Real-Time PCR System. MiR-133a expression was quantified in relation to the expression of small nuclear U6 RNA.

### Statistical analysis

Data are presented as the mean ±SD. The differences between two groups were assessed using Student's *t*-test, and the Kaplan-Meier method was used to estimate overall survival. Differences in survival between two groups were analyzed using the log-rank test. Multivariate Cox proportional hazard regression analysis with stepwise selection was used to evaluate independent prognostic factors associated with patient survival, and the expression of miR-133a, age, gender, tumor stage, and histology were used as covariates. All analyses were performed with SAS version 9.1 software (SAS, SAS institute, Cary, NC). Two-tailed tests were used, and *P* values <0.05 were considered to indicate statistical significance.

## Results

### MiR133a is downregulated in malignant lung cancer cells and inhibits the cell invasive capacity

**Figure 1:**
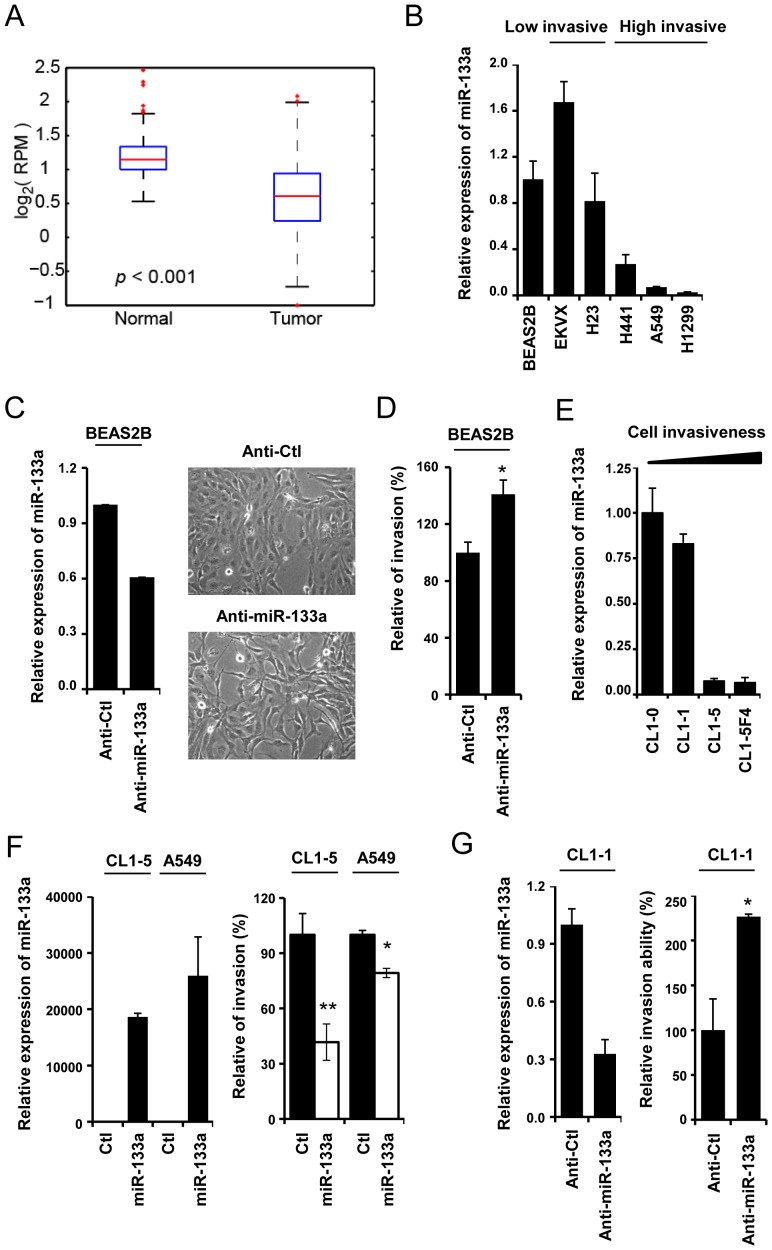
The miR-133a tumor suppressor modulates cell invasive capacity in lung cancer. (A) The expression levels of miR-133a in 434 tumor tissues and 46 normal tissues from the TCGA lung adenocarcinoma database were examined. (B) The expression level of endogenous miR-133a in BEAS-2B, EKVX, H23, H441, A549 and H1299 cell lines. MiR-133a expression level (C, left panel), as measured by real time RT-PCR, and cell morphology (C, right panel) in BEAS-2B cells were examined 72 hours after transfection of the anti-miR-133a inhibitor. (D)The number of invasive cells was counted 24 hours after seeding cells that post-treated anti-miR-133a inhibitor (100 nM) for 48 hours in a transwell containing matrigel. (E) The endogenous level of miR-133a in CL1-0, CL1-1, CL1-5 and CL1-5F4 cells. (F) Measurement of the invasion in CL1-5 and A549 cells transiently infected with the AS2-Neo (Ctl) or AS2-Neo-miR-133a-expressing viruses (right panel). The expression level of miR-133a was normalized to that of RNU48, which was used as an internal control (left panel) (G) 48 hours after transient transfection of the anti-miR-133a inhibitor, the invasive capacity of CL1-1 cells was determined (right panel). The expression level of miR-133a was normalized to that of RNU48, which was used as an internal control (left panel).

In the TCGA lung adenocarcinoma database (https://tcga-data.nci.nih.gov/tcga/tcgaHome2.jsp), we found significantly reduced expression levels of miR-133a in 434 tumor tissues compared with 46 normal lung tissues (Figure 1A). To further evaluate the role of miR-133a during lung cancer progression, the expression level of miR-133a in transformed normal bronchial epithelium cells (BEAS-2B cells) and five lung cancer cell lines with different invasive capacities was examined. We found that the expression levels of miR-133a were higher in BEAS-2B cells and in cells with low invasiveness (EKVX and H23) [23] than in those with high invasive ability (A549, H441 and H1299) [24] (Figure 1B). These data suggest that miR-133a is dysregulated in lung cancer and that the expression of miR-133a negatively correlates with cell invasiveness.

To further examine whether miR-133a is associated with cancer progression, we suppressed the expression of miR-133a by an anti-miR-133a inhibitor in BEAS-2B cells and examined cell morphology and invasiveness. Inhibition of miR-133a expression increased the population of spindle-shaped cells and enhanced BEAS-2B invasive ability (Figure 1C and 1D), implying that reduced miR-133a expression in transformed lung bronchial epithelial cells may enhance the invasive phenotype.

We further examined miR-133a expression using a panel of well-established lung cancer sub-lines, namely CL1-0, CL1-1, CL1-5 and CL1-5F4, with increasing invasive capacity [22]. We found that the expression of miR-133a gradually decreased as the invasion ability increased (Figure 1E). Next, we manipulated the expression of miR-133a in high invasive cancer cell lines, namely CL1-5 and A549, and ectopic expression of miR-133a decreased their invasive capacities (Figure 1F, right panel). Conversely, treatment with the miR-133a inhibitor increased the number of invasive cells in CL1-1 cells (Figure 1G, right panel). Thus, these results suggest that miR-133a governs cell invasiveness in lung cancer cells. In addition, cell proliferation of CL1-5 and A549 were decreased in miR-133a-expressing cells (Figure S1A), suggesting that miR-133a may inhibit growth in these lung cancer cells.

### Multiple oncogenic receptors are directly regulated by miR-133a

**Figure 2:**
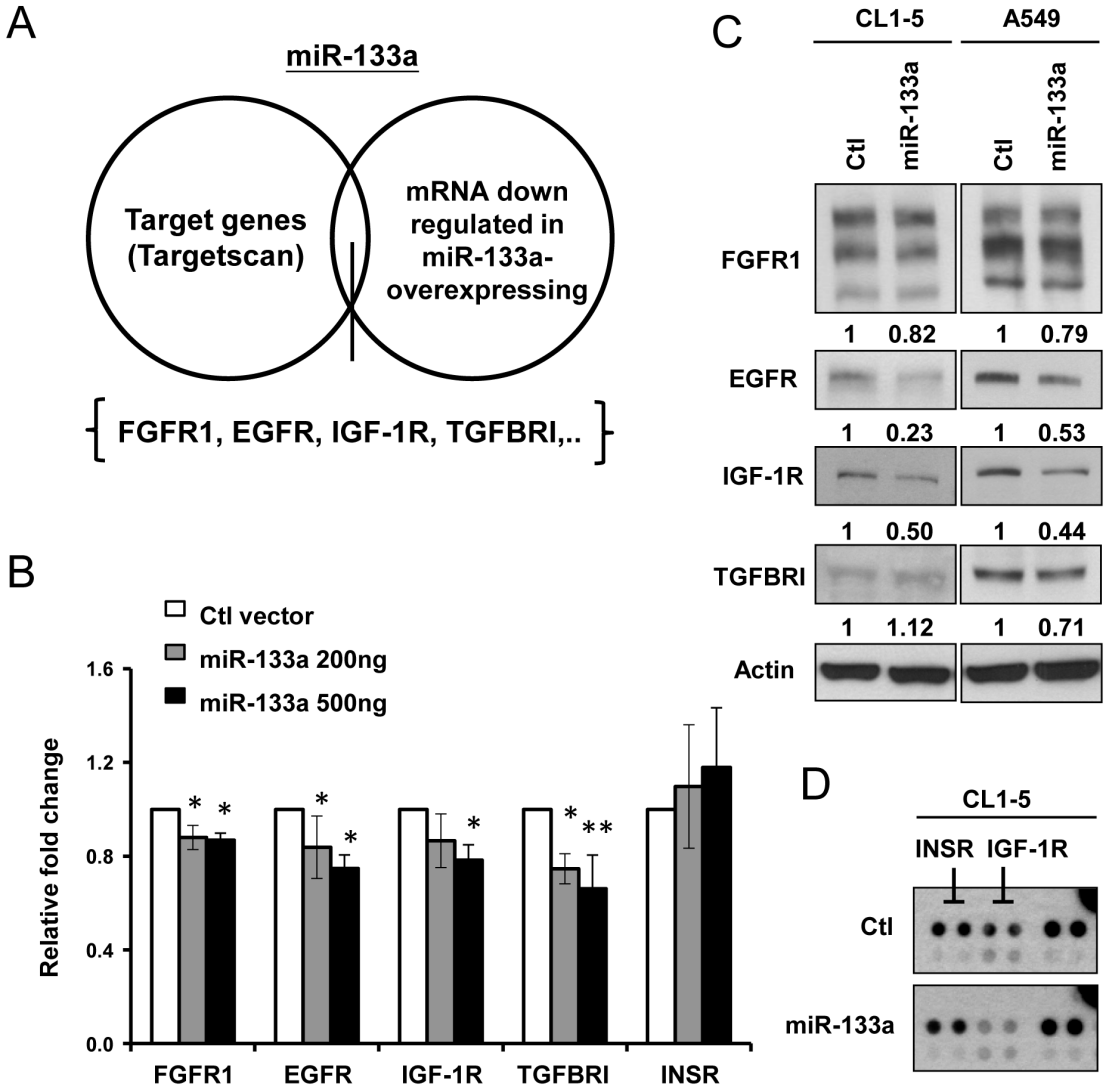
Oncogenic receptor expression and activation are regulated by miR-133a in lung cancer cell lines. (A) The target genes of miR-133a were determined from Targetscan prediction software and the two database containing mRNA expression levels of receptors in miR-133a-expressing cells. (B) Co-transfection of CL1-0 cells with the AS2-Neo vector (Ctl) or AS2-Neo-miR-133a-expressing plasmid with firefly luciferase fused with 3′UTR sequences of putative miR-133a target genes. Luciferase activity was measured, and the relative ratio of the activity in the miR-133a groups to that in the control vector group is presented. (C) 72 hours after transient infection with the miR-133a-expressing virus, the protein expression levels of receptors in CL1-5 and A549 cells were determined. (D) The phosphorylation level of IGF-1R was determined in CL1-5/AS2-Neo- or CL1-5/miR-133a-expressing cells by the Phospho-Receptor Tyrosine Kinase Array.

To investigate the mechanism of miR-133a-mediated suppression of cancer cell invasion, we used TargetScan (http://www.targetscan.org/) to predict miR-133a downstream targets. Among them, a target network containing several receptor tyrosine kinases (RTKs), such as FGFR1, EGFR, IGF-1R, insulin receptor (INSR), and a serine/threonine kinase receptor, TGFBR1, are presented as possible candidates, all of which possess several putative miR-133a binding sites at their 3′- untranslated regions (3′-UTR) (Figure S2A). We further investigated the mRNA levels of these receptors in miR-133a-overexpressing cancer cell lines by combining the microarray database (http://www.ncbi.nlm.nih.gov/geo/query/acc.cgi?acc=GSE20028 and http://www.ncbi.nlm.nih.gov/geo/query/acc.cgi?acc=GSE26032) (Figure S2B). The mRNA levels were consistent with the *in silico* prediction results, suggesting that FGFR1, EGFR, IGF-1R and TGFBR1 may be downregulated by miR-133a (Figure 2A). To evaluate whether miR-133a directly regulates the expression of these receptors, the 3-′UTRs of these receptors containing the predicted miR-133a target sites were cloned into the luciferase reporter plasmid for a reporter assay. The luciferase activity of the constructs containing the 3′UTRs of EGFR, FGFR1, IGF-1R, TGFBR1 were significantly reduced in the presence of miR-133a, although the fold change in FGFR1 was not as dramatic as the others, and the luciferase activity of INSR was not affected by miR-133a (Figure 2B). We further evaluated whether TGFBR1 may be directly regulated by miR-133a since this correlation has never been reported. The luciferase activity was decreased in the presence of miR-133a, but this repression was alleviated by the addition of an anti-miR-133a inhibitor (Figure S3A). These results indicate that miR-133a may regulate the expression of FGFR1, EGFR, IGF-1R, and TGFBR1.

To test whether FGFR1, EGFR and IGF-1R-mediated signaling pathways are activated in CL1-5 and A549 cell lines, we detected their activating levels by phospho-RTK proteome assays and found that EGFR and IGF-1R, but not FGFR1, were activated in these cells (Figure S4A). To investigate whether miR-133a regulates the endogenous protein expressions of these receptors, we transduced CL1-5 and A549 cells with miR-133a-expressing viruses and detected the protein levels of these receptors. As shown in Figure 2C, the expressions of EGFR and IGF-1R were decreased by the ectopic expression of miR-133a in CL1-5 and A549 cells (Figure 2C). Downregulation of TGFBR1 expression was observed in A549 cells but not in CL1-5 cells where the endogenous level of TGFBR1 was low (Figure 2C). The FGFR1 protein level was mildly downregulated, which corresponded to the luciferase results (Figure 2C). Moreover, the ectopic expression of miR-133a decreased the IGF-1R activity in CL1-5 cells (Figure 2D). These results demonstrate that miR-133a may down-regulate the EGFR, IGF-1R and TGFBR1 to different degrees in lung cancer cells.

### Knockdown of IGF-1R or TGFBR1 inhibits lung cancer invasion and proliferation through AKT-mediated signaling

**Figure 3:**
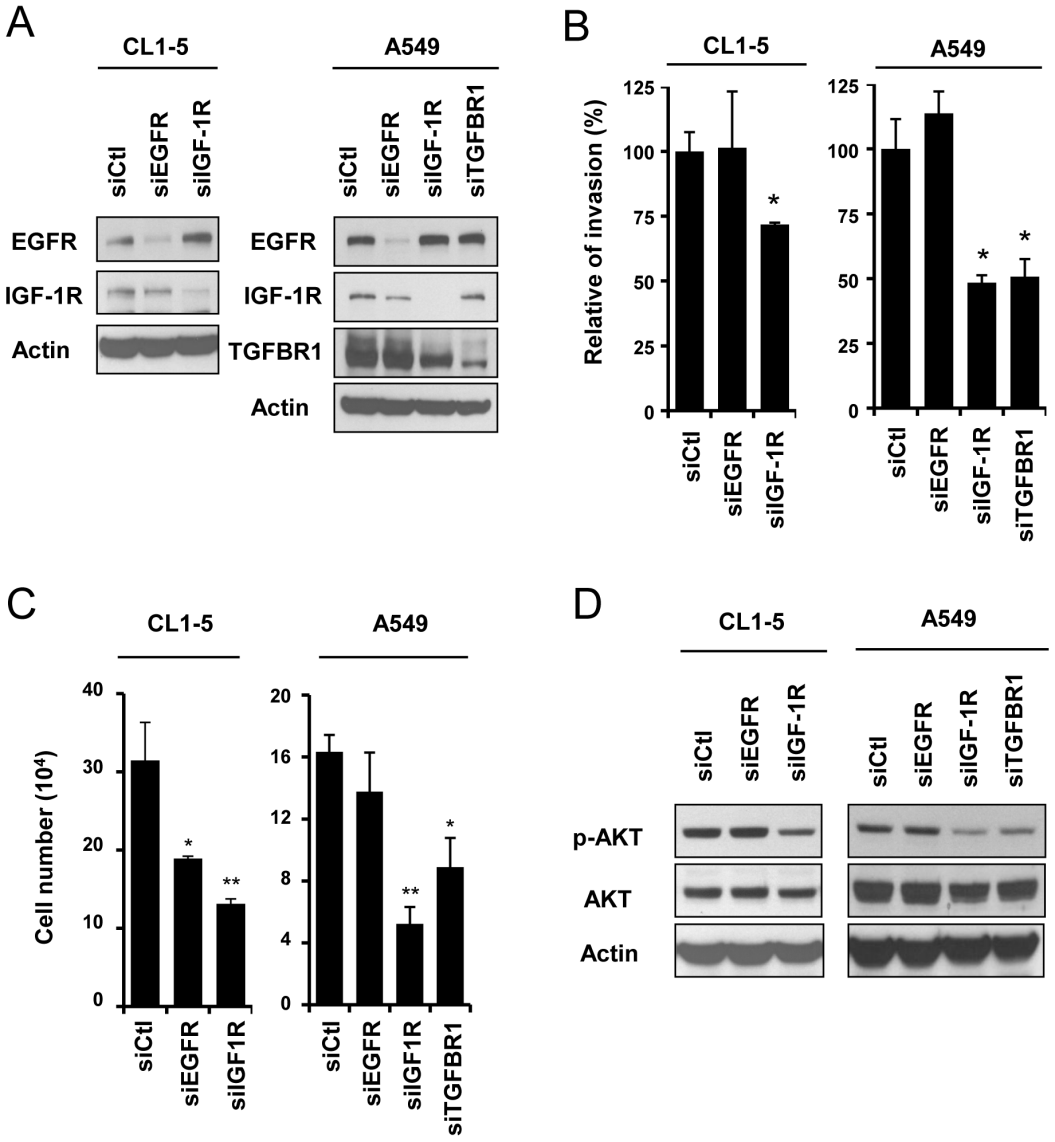
IGF-1R and TGFBR1 mediate cell invasion through AKT signaling in lung cancer cell lines. (A) Measurement of the protein levels of receptors CL1-5 or A549 cell transfected with siEGFR, siIGF-1R or siTGFBR1. (B) Measurement of the cell invasion ability of CL1-5 or A549 cells transfected with siEGFR, siIGF-1R or siTGFBR1 for 48 hours. The number of invasive cells was counted 20 hours after the transfected cells were seeded and was presented relative to invasion of cells transfected with siCtl. (C) Measurement of the cell proliferation ability of CL1-5 or A549 cells transfected with siEGFR, siIGF-1R or siTGFBR1 for 72 hours. (D) Representative immunoblots show protein levels of pAKT (Ser473), AKT and β-actin in these cells.

Since we identified that miR-133a could suppress cell invasive and proliferative abilities, we further evaluated whether EGFR, IGF-1R and TGFBR1 could modulate these functions in CL1-5 and A549 cells by the receptors specific siRNA-silencing approach (Figure 3A). Forty-eight hours after siRNA transfection, downregulation of IGF-1R decreased the number of invading and proliferating cells in both CL1-5 and A549 cells (Figure 3A, 3B and 3C). In addition, TGFBR1-silenced A549 cells also reduced invasive ability and cell growth compared to the control cells (Figure 3B and 3C). Furthermore, knockdown of EGFR expression induced a morphological change similar to the miR-133a-expressing phenotype (Figure S5A), and decreased cell proliferation in CL1-5 cells (Figure 3C). However, EGFR knockdown did not influence cell invasiveness in neither cell lines (Figure 3B). These results indicate that IGF-1R and TGFBR1 control cell invasiveness and cell proliferation while EGFR governs the latter solely, which raises the possibility that EGFR may activate a different downstream signaling pathway from IGF-1R/TGFBR1 in CL1-5 and A549 cells.

One of the main downstream signals of these receptors is the activation of AKT, which acts as a key regulator in cancer progression by promoting cell growth, anti-apoptotic effects, and cell invasion [25], and these regulations can be detected in CL1-5 (Figure S6A) in A549 [26], [27] cell lines. To test whether AKT-mediated signal is involved in the cell lines used in the present study, we first detected the phosphorylation status of AKT by knocking down the expression levels of each receptor (Figure 3D). Phosphorylated AKT was reduced when IGF-1R and TGFBR1 were knocked down in CL1-5 and A549 cells (Figure 3D). Interestingly, inhibition of EGFR did not affect the AKT activity in these cells (Figure 3D). These results indicate that IGF-1R and TGFBR1 may control cell invasiveness and cell proliferation through the activation of an AKT-mediated signaling.

### MiR-133a regulates AKT activity through suppressing downstream targets of IGF-1R and TGFBR1

**Figure 4:**
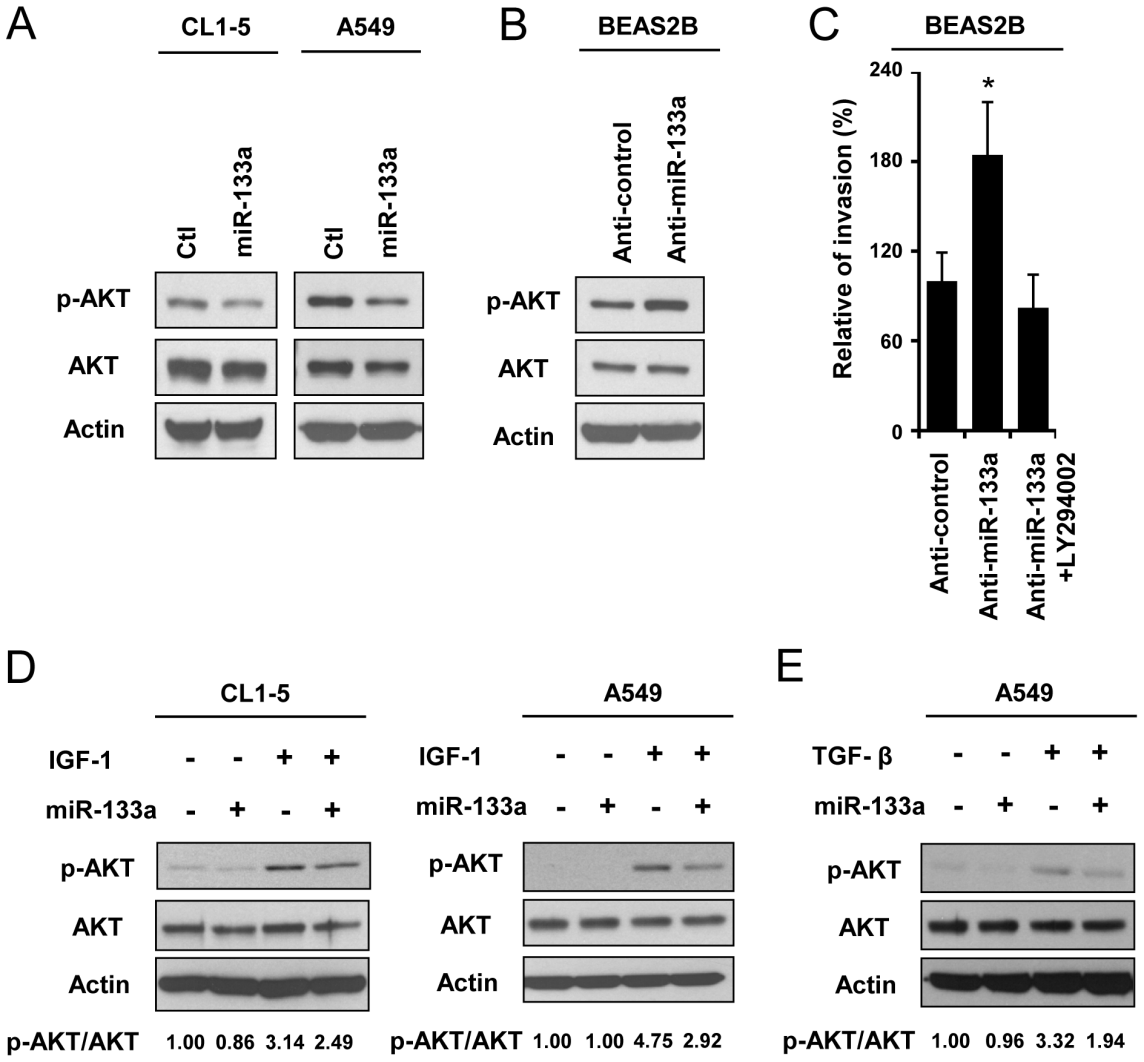
MiR-133a modulates AKT activation and suppresses IGF-1- and TGF-β-mediated AKT signaling in lung cancer cell lines. (A) 72 hours after infection with the AS2-neo (Ctl) or AS2-Neo-miR-133a viruses, the protein levels of pAKT (Ser473), AKT and β-actin were examined in CL1-5 and A549 cells. (B) 72 hours after transfection with the anti-miR-133a inhibitor, the protein levels of pAKT (Ser473), AKT and β-actin were examined in BEAS-2B cells. (C) Measurement of the cell invasion ability of BEAS-2B cells after anti-miR-133a inhibitor (100 nM) and LY294002 (50 µM) pre-treated for 48 hours. The number of invasive cells was counted 24 hours after seeding cells in a transwell containing matrigel. (D,E) AKT phosphorylation levels of stable miR-133a-expressing CL1-5 and A549 cells or control CL1-5 and A549 cells were examined after treatment with 250 ng/ml IGF-1 for 30 min (CL1-5 cells) or 2.5 min (A549 cells) (D) or treatment with 0.2 ng/ml TGF-β for 5 min (A549 cells) (E). The quantification of the immunoblot data are expressed as a pAKT (Ser473)/AKT ratio after normalization to β-actin.

To evaluate whether miR-133a regulates AKT activity, we ectopically expressed miR-133a in CL1-5 and A549 cells. Overexpression of miR-133a decreased AKT phosphorylation in both CL1-5 and A549 cells (Figure 4A). We then suppressed miR-133a expression by using an anti-miR-133a inhibitor and found that inhibition of miR-133a enhanced AKT activation in BEAS-2B cells (Figure 4B). To further examine whether miR-133a suppresses invasion through AKT signaling, we treated BEAS-2B cells with the miR133a inhibitor and PI3K/AKT inhibitor, LY294002 (Figure S7A). Increased cell invasiveness by miR-133a inhibition was abrogated in the presence of LY294002 (Figure 4C). To validate whether miR-133a inhibits AKT signaling through repressing IGF-1R and TGFBR1, we next treated cells with their ligands. In the presence of IGF-1, the AKT phosphorylation level was reduced in miR-133a-overexpressing CL-1-5 and A549 cells (Figure 4D). In addition, TGF-β-induced AKT phosphorylation was also reduced in the miR-133a-overexpressing A549 cells (Figure 4E). These results show that miR-133a regulates IGF-1- and TGF-β-mediated downstream signaling through downregulating their receptors.

### MiR-133a suppresses cancer metastasis *in vivo*

**Figure 5:**
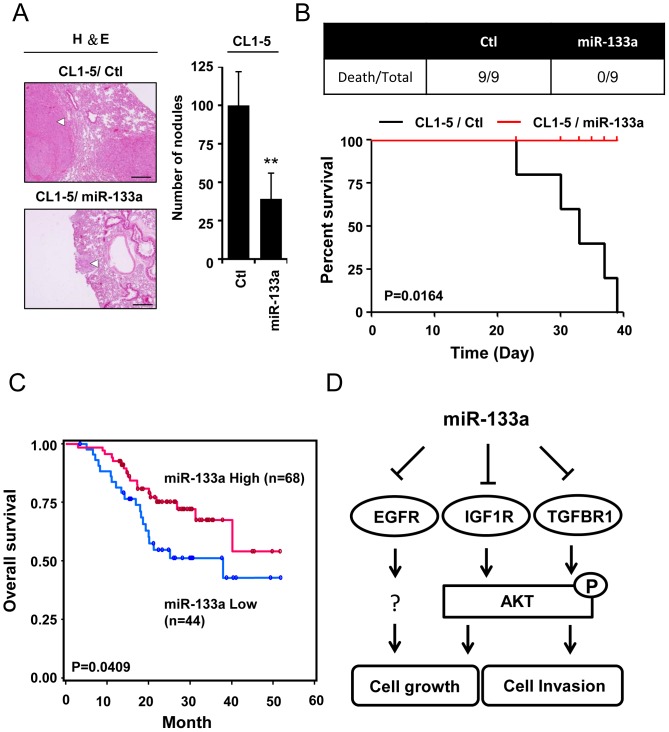
MiR-133a expression decreases cancer metastasis *in vivo* and associates with longer survival rates in NSCLC patients. (A) Effects of miR-133a in metastasis *in vivo*. Left panel: histological examinations of the lung tumors by hematoxylin-eosin (H&E) staining. Representative lungs of mice intravenously (i.v.) injected with CL1-5/Ctl, CL1-5/miR-133a. White arrows indicate the lung tumors. Scale bars, 200 µm. Right panel: Numbers of metastatic tumor nodules were calculated 39 weeks after tail-vein injection (n = 9 per group). (B) The number of death/total (top panel) and survival curve (bottom panel) in each group at 39 days after i.v. injection were presented. Data were expressed as the mean ±SD by the log-rank (Mantel-Cox) test. P = 0.0164 (C) Kaplan-Meier plots of overall survival in 112 NSCLC patients in high- and low-risk groups based on miR-133a expression levels (P = 0.0409). (D) Model showing that miR-133a modulates the receptor-mediated cancer malignancy in NSCLC.

To investigate whether the expression of miR-133a inhibits cancer metastasis *in vivo*, CL1-5/Ctl and CL1-5/miR-133a cells were inoculated directly into the circulation of mice. Mice with CL1-5/Ctl cells developed more pulmonary nodules than those injected with CL1-5/miR-133a cells (mean number of nodules: 99.88±21.93 for line Ctl and 39.11±16.76 for line miR-133a, p<0.01) (Figures 5A). Similarly, the mortality of mice with CL1-5/Ctl cells was higher than that of mice with CL1-5/miR-133a cells, whereas all mice with miR-133a were still alive at 39 days after tumor injection (Figure 5B). Therefore, the pulmonary metastasis and survival analysis of the CL1-5 murine model support the findings that miR-133a expression could suppress metastasis *in vivo*.

### MiR-133a acts as a prognostic indicator of the clinical outcome

Because miR-133a suppresses multiple oncogenic receptors and inhibits lung cancer cell invasiveness, we explored the potential biological significance of miR-133a expression in lung cancer progression. We evaluated the correlation between the miR-133a expression profile and the overall survival in tumor specimens from 112 lung cancer patients. The characteristics of the 112 patients are provided in Table S1, and miR-133a levels were measured by real-time RT-PCR. Kaplan-Meier analysis showed that low expression levels of miR-133a were significantly associated with poor overall survival (P = 0.0409; Figure 5C). Multivariable Cox regression analyses were used to evaluate the associations of various independent prognostic factors with patients' survival (Table 1). The results showed that the phenotype of high miR-133a expression was a protective factor when age, gender, and tumor histological type were considered (hazard ratio: 0.455, 95% CI: 0.233-0.888; p = 0.0209) (Table 1). This result suggested that patients who have tumors with higher miR-133a expression levels may have better clinical outcome.

**Table 1:** Multivariable Cox regression analysis of miR-133a levels and overall survival in 112 NSCLC patients.

Variable	Hazard ratio (95% C.I.)	p-value
miR-133a	0.455 (0.233–0.888)	0.0209
Gender	0.711 (0.307–1.647)	0.426
Age	1.057 (1.018–1.097)	0.0038
Stage	2.5 (1.678–3.726)	<0.0001

## Discussion

Metastasis is one of the most critical hallmarks of cancer, especially in lung cancer. There is an urgent need for the identification of metastatic-related factors and their underlying molecular mechanisms. MiRNAs play an important role in tumorigenic and metastatic progression, and they are involved in the pathogenesis and prognosis of NSCLC. Studies of prognostic markers in lung cancer have indicated that let-7-a and miR-221 expression levels correlate with an improved survival [28] and that miR-34a and miR-155 expression levels correlate with lymphoid infiltration and a poor survival [29]. Here, we suggested that miR-133a may serve as a prognostic biomarker and that its expression level positively correlates with the overall survival in NSCLC patients. We have also found that miR-133a modulates cancer invasiveness and the proliferation ability in lung cancer cells. We also identified that miR-133a can downregulate multiple targets and that these targets are all oncogenic membrane receptors, namely EGFR, IGF-1R, and TGFBR1 (Figure 5D). The oncogenic roles of these receptors have been well identified, and they are all important therapeutic targets or molecular markers for cancer treatment [30], [31].

MiR-133a is a key regulator of cardiomyogenesis and acts as a tumor suppressor in multiple cancer malignancies [32], [33]. However, the molecular mechanism of miR-133a-involved lung cancer progression has not been clarified. Here, we found that miR-133a functions as a tumor suppressor and that its expression level is significantly downregulated in NSCLC cells, and this phenomenon was observed even in highly invasiveness cells. We found that miR-133a can suppress cancer cell invasion, metastasis and proliferation abilities, and we also found that inhibition of IGF-1R and TGFBR1 reduces both cell invasion and cell proliferation. In addition, ectopic expression of miR-133a in highly invasive cell lines decreases IGF-1R- and TGFBR1-mediated AKT activation, thereby implying that miR-133a may modulate cell invasion through downregulating IGF-1R- and TGFBR1-mediated AKT signaling. Although previous studies have indicated that miR-133a can inhibit cell invasion and proliferation through downregulating EGFR [34], our *in vitro* lung cancer systems show that EGFR expression does not affect the invasion ability in CL1-5 and A549 cells. However, there is a miR-133a-induced morphological change similar to the EGFR-knockdown phenotype (Figure S5A). In addition to a decrease in invasiveness, ectopic expression of miR-133a reduces the cell proliferation activity in CL1-5 and A549 cells (Figure S1A). In lung cancer, EGFR overexpression is associated with cancer proliferation [35] and downregulated EGFR causes cell growth inhibition in CL1-5 cells (Figure 3C). These results suggested that miR-133a may modulate EGFR-mediated proliferation ability through regulating other signaling pathway. In summary, we propose that miR-133a can regulate oncogenic receptor-related cell invasiveness and proliferation in NSCLCs (Figure 5D).

IGF-1R and INSR belong to the IGF signaling pathway, which plays central roles in cell growth, differentiation, survival, cell transformation and cancer metastasis [8], [36]. Unlike INSR expression, which contributes less to tumorigenesis, the malignancy of dysregulated IGF-1R in cancer progression has been investigated, and targeting IGF-1R in cancer treatment is currently under development. However, the results of recent clinical trials with IGF-1R inhibitors have encountered several hindrances, which need to be resolved. For instance, due to the homology between IGF-1R and INSR, the inhibition of IGF-1R signaling disrupts INSR signaling and results in unwanted side effects, such as hyperglycemia and hyperinsulinemia. In this study, we noticed that both INSR and IGF-1R contain the miR-133a target sequence in the 3′UTR. However, miR-133a specifically targets IGF-1R and reduces its receptor activity while INSR is not affected. Therefore, miR-133a mimics may be promising small compounds in regulating IGF-1R signaling in tumor cells.

In this study, we identified TGFBR1 as a novel downstream target of miR-133a. However, TGF-β signaling has dual functions in cancer progression [9]. TGF-β has a potent growth inhibitory effect on epithelial and lymphoid tissues at the early stage of tumor development, but it also becomes a pro-oncogenic factor that stimulates tumor cell growth and invasiveness at the later stage [9]. We observed that the A549/TGFBR1-silenced cells present an epithelial-like morphology with reduced cell invasive and proliferative ability, suggesting that TGFBR1 has the oncogenic property in highly invasive cells. Both miR-133a overexpression and TGFBR1 silencing leads to AKT activation, and miR-133a-overexpressing cells have reduced TGF-β/TGFBR1-mediated AKT signaling, thereby indicating that miR-133a may have the potential role to regulate TGF-β-mediated EMT.

Previous studies have indicated that miR-133b-regulated FGFR1-mediated tumor growth occurs in gastric cancer [37]. Although our results showed that functional FGFR1 signaling is not significantly changed in our lung adenocarcinoma cell system, FGFR1 suppression by miR-133a is detected in the reporter assay (Figure 2B), and miR-133a-suppressed FGFR1 mRNA levels have been detected in four squamous cell carcinomas (Figure S2B). In NSCLC patients, FGFR1 amplifications regulates cell proliferation through activation of the MAPK and PI3K/AKT pathways in about 20% of squamous cell carcinomas but they are rarely detected in adenocarcinomas [4]. Therefore, although FGFR1 mediated regulation has not been detected in CL1-5 and A549 cells, it may be an important factor in miR-133a-mediated tumor suppression in squamous cell carcinoma tumorigenesis.

In summary, the microRNA, miR-133a, inhibits the expression levels of multiple oncogenic receptors, i.e., EGFR, IGF1R, and TGFBR1, and mediates cell growth and invasion in lung adenocarcinoma. Moreover, miR-133a expression levels predict the clinical outcome in NSCLC, thereby serving as a suitable prognostic factor. Therefore, miR-133a is a promising theranostic agent, and the development of miR-133a mimics should possess a great potential for cancer treatment.

## Supporting Information

Figure S1 **Cell proliferation of miR-133a-overexpressing CL1-5 and A549 cells.** (A) Measurement of cell proliferation of miR-133a-overexpressing CL1-5 and A549 cells. 72 hours after transient infection with AS2-Neo (Ctl) or AS2-Neo-miR-133a-expressing viruses, the cells (1×10^4^) were seeded in 6-well plates, and the cell numbers were counted on the indicated days. Each conditions were performed in triplicate.
(TIF)

Figure S2 **FGFR, EGFR, IGF-1R and TGFBR1 are direct targets of miR-133a.** (A) Putative miR-133a binding sites of the five receptors, including FGFR, EGFR, IGF-1R, INSR and TGFBR1, were identified by computational algorithms from Targetscan (a–e). (B) Fold changes of FGFR, EGFR, IGF-1R and TGFBR1 were estimated from expression profiles (Data set: GSE20028 and GSE26032) of miR-133a-transfected cancer cells vs. control cells. SAS and HSC3 were the oral squamous cell lines; H157 and PC10 were the lung squamous cell carcinoma cell lines.
(TIF)

Figure S3 **TGFBR1 3′UTR is suppressed by miR-133a.** (A) Co-transfection of CL1-5 cells with AS2-Neo vector (Ctl) or AS2-Neo-miR-133a-expressing plasmid with firefly luciferase fused with 3′UTR of TGFBR1 for 6 hours and then incubated with anti-miR-Ctl or an anti-miR-133a inhibitor (100 nM) in complete medium for 36 hours. Luciferase activity was measured, and the relative ratio of the activity in the miR-133a groups to that in the control vector group is presented.
(TIF)

Figure S4 **Phospho-receptor detection in CL1-5 and A549 cell lines.** (A) CL1-5 and A549 cell lysates were incubated with nitrocellulose membranes that were conjugated with phospho-receptor antibodies in duplicate. The phosphorylation levels of EGFR, FGFR, INSR and IGF-1R were determined.
(TIF)

Figure S5 **Cell morphology of miR-133a-overexpressing or oncogenic receptor-silenced CL1-5 or A549 cells.** The cell morphology of CL1-5 (A) or A549 (B) was determined 72 hours after transient infection with AS2-Neo (Ctl) or AS2-Neo-miR-133a-expressing viruses (upper panel) or 48 hours after transient transfection with siCtl, siEGFR, siIGF-1R or siTGFBR1 treatment (middle and bottom panel, respectively).
(TIF)

Figure S6 **AKT signaling is essential for cell proliferation and cell invasion in CL1-5 cell lines.** (A) CL1-5 cells were pre-treated with the AKT inhibitor (7.5 µM) for 24 hours, and then seeded into chambers with AKT-inhibitor containing medium. The invasive cells were determined 20 hours post-incubation. (B) The cell numbers of CL1-5 were counted after AKT inhibitor treatment for 3 days.
(TIF)

Figure S7 **Up-regulation of phospho-AKT mediated by an anti-miR-133a inhibitor can be reduced by the PI3K/AKT inhibitor in BEAS-2B cells.** (A) Representative immunoblots showing the protein levels of pAKT (Ser473), AKT and β-actin in BEAS-2B cells after treatment with an anti-miR-133a inhibitor (100 nM) with either DMSO or LY294002 (50 µM) for 48 hours.
(TIF)

Table S1 **MiR-133a expression in relation to clinical parameters and pathological characteristics.** The clinical characteristics of the 112 patients with NSCLC are summarized.
(DOCX)

Materials and Methods S1 **Luciferase reporter assay with anti-miR-133a treatment.** One day before transfection, CL1-5 cells were seeded in 12-well plates at a concentration of 6×10^4^ per well. Next, 200 ng of the pLKO-AS2 neo vector or pLKO-AS2 miR-133a plasmid was co-transfected with 50 ng of pGL3-TGFBR1-3′UTR. The Renilla luciferase plasmid (pRL-TK, Promega, Madison, WI) was co-transfected as a transfection control. Six hours post-transfection, cells were treated with an anti-miR-Ctl or anti-miR-miR-133a inhibitor (100 nM). Cells were lysed 36 hours post-transfection, and luciferase activity was measured using a Dual-Luciferase system (Promega, Madison, WI) according to the manufacturer's protocol.
(DOCX)
