# Supplementary figures and images for: MicroRNA-133a Suppresses Multiple Oncogenic Membrane Receptors and Cell Invasion in Non-Small Cell Lung Carcinoma
Source: PLoS One. 2014 May 9;9(5):e96765. doi: 10.1371/journal.pone.0096765 (PMC4016005; doi:10.1371/journal.pone.0096765)

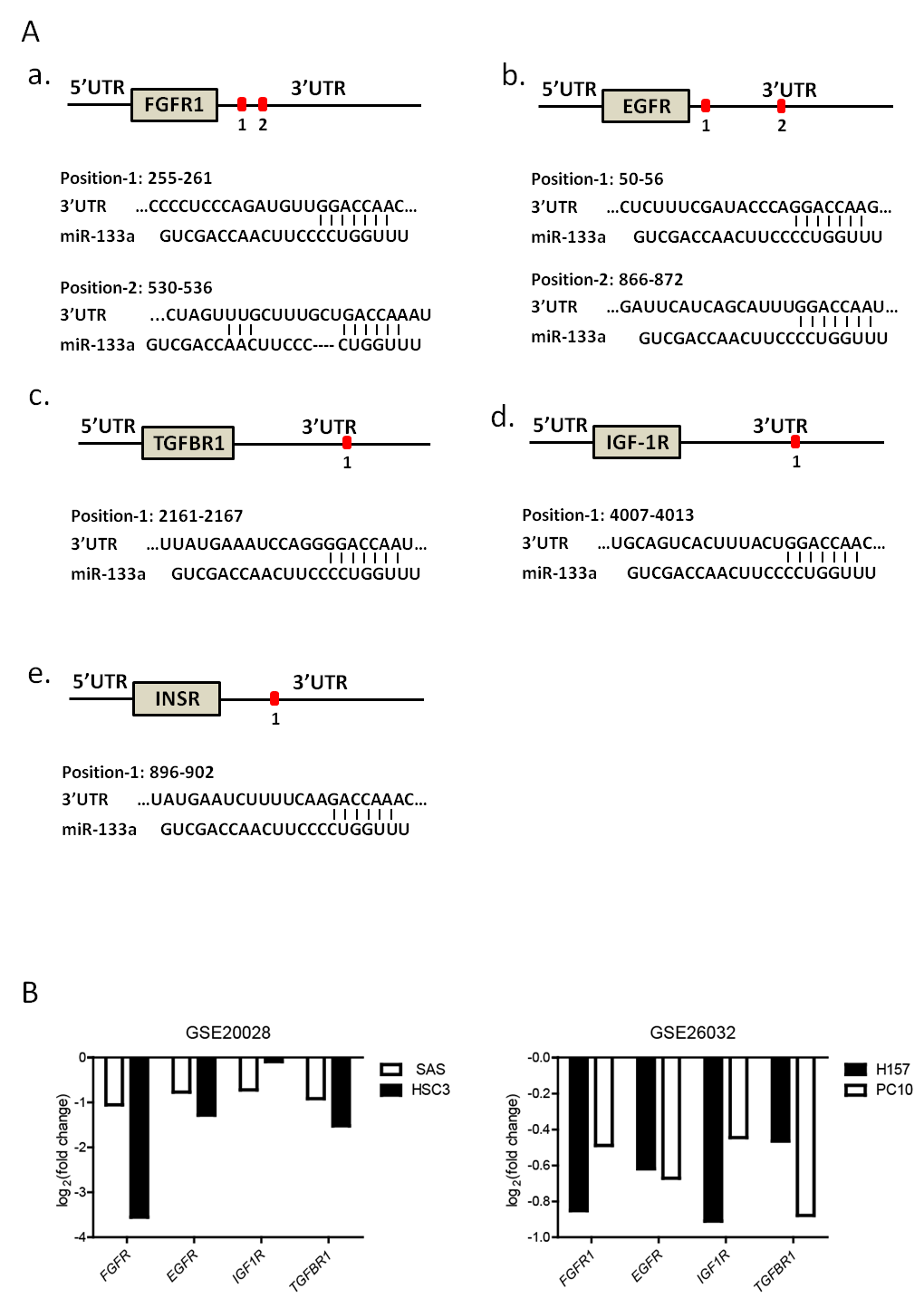

Supplement: Figure S2 — FGFR, EGFR, IGF-1R and TGFBR1 are direct targets of miR-133a. (A) Putative miR-133a binding sites of the five receptors, including FGFR, EGFR, IGF-1R, INSR and TGFBR1, were identified by computational algorithms from Targetscan (a–e). (B) Fold changes of FGFR, EGFR, IGF-1R and TGFBR1 were estimated from expression profiles (Data set: GSE20028 and GSE26032) of miR-133a-transfected cancer cells vs. control cells. SAS and HSC3 were the oral squamous cell lines; H157 and PC10 were the lung squamous cell carcinoma cell lines. (TIF) [file pone.0096765.s002.tif]
