# Supplementary material for: MicroRNA-133a Suppresses Multiple Oncogenic Membrane Receptors and Cell Invasion in Non-Small Cell Lung Carcinoma
Source: PLoS One. 2014 May 9;9(5):e96765. doi: 10.1371/journal.pone.0096765 (PMC4016005; doi:10.1371/journal.pone.0096765)
Supplement: Table S1 — MiR-133a expression in relation to clinical parameters and pathological characteristics. The clinical characteristics of the 112 patients with NSCLC are summarized. (DOCX) [file pone.0096765.s008.docx]

**Table S1. miR-133a expression in relation to clinical parameters and pathological characteristics**

|  |  |  | **miR-133a** | | |  |
| --- | --- | --- | --- | --- | --- | --- |
| **Category** | **Subcategory** | **All** | **Low (%)** | | **High (%)** | **P** |
| **Age mean(SD)** |  | **65.88 (12.17)** | **65.82(9.40)** | | **65.92(13.74)** | **0.963^a^** |
| **Cell type** | ***Adenocarcinoma*** | **55 (49.11)** | **23 (52.27)** | | **32 (47.06)** | **0.478^b^** |
|  | ***Large cell carcinoma*** | **7 (6.25)** | **4 (9.09)** | | **3 (4.41)** |  |
|  | ***Squamous cell carcinoma*** | **50 (44.64)** | **17 (38.64)** | | **33 (48.53)** |  |
| **Gender** | ***Male*** | **88 (78.57)** | **35 (79.55)** | | **53 (77.94)** | **1.00^b^** |
|  | ***Female*** | **24 (21.43)** | **9 (20.45)** | | **15 (22.06)** |  |
| **Tumor stage** | **Stage *I*** | **47 (41.96)** | **17 (38.64)** | | **30 (44.12)** | **0.804^b^** |
|  | **Stage *II*** | **28 (25.00)** | **11 (25.00)** | **17 (25.00)** | |  |
|  | **Stage *III*** | **37 (33.04)** | **16 (36.36)** | **21 (30.88)** | |  |

^a^ p-value used t-test to calculate, ^b^ p-value used fisher’s exact test to calculate.
