# Supplementary material for: MicroRNA-133a Suppresses Multiple Oncogenic Membrane Receptors and Cell Invasion in Non-Small Cell Lung Carcinoma
Source: PLoS One. 2014 May 9;9(5):e96765. doi: 10.1371/journal.pone.0096765 (PMC4016005; doi:10.1371/journal.pone.0096765)
Supplement: Materials and Methods S1 — Luciferase reporter assay with anti-miR-133a treatment. One day before transfection, CL1-5 cells were seeded in 12-well plates at a concentration of 6×10^4^ per well. Next, 200 ng of the pLKO-AS2 neo vector or pLKO-AS2 miR-133a plasmid was co-transfected with 50 ng of pGL3-TGFBR1-3′UTR. The Renilla luciferase plasmid (pRL-TK, Promega, Madison, WI) was co-transfected as a transfection control. Six hours post-transfection, cells were treated with an anti-miR-Ctl or anti-miR-miR-133a inhibitor (100 nM). Cells were lysed 36 hours post-transfection, and luciferase activity was measured using a Dual-Luciferase system (Promega, Madison, WI) according to the manufacturer's protocol. (DOCX) [file pone.0096765.s009.docx]

**Supporting Information**

**Materials and Methods**

Luciferase reporter assay with anti-miR-133a treatment

One day before transfection, CL1-5 cells were seeded in 12-well plates at a concentration of 6 × 10^4^ per well. Next, 200 ng of the pLKO-AS2 neo vector or pLKO-AS2 miR-133a plasmid was co-transfected with 50 ng of pGL3-TGFBR1-3’UTR. The Renilla luciferase plasmid (pRL-TK, Promega, Madison, WI) was co-transfected as a transfection control. Six hours post-transfection, cells were treated with an anti-miR-Ctl or anti-miR-miR-133a inhibitor (100 nM). Cells were lysed 36 hours post-transfection, and luciferase activity was measured using a Dual-Luciferase system (Promega, Madison, WI) according to the manufacturer’s protocol.

**Figure legends**


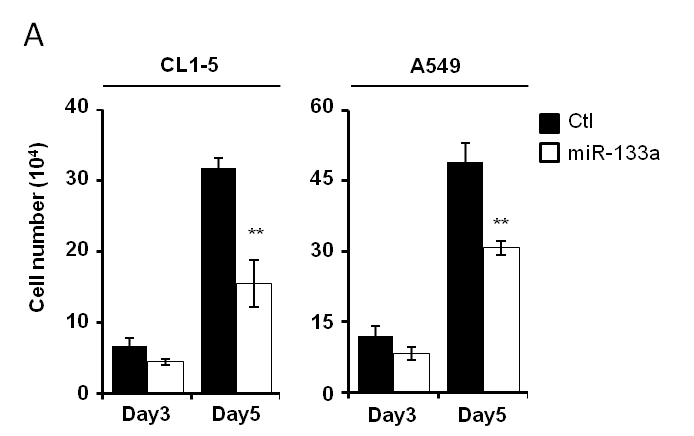


Figure S1. Cell proliferation of miR-133a-overexpressing CL1-5 and A549 cells.

(A) Measurement of cell proliferation of miR-133a-overexpressing CL1-5 and A549 cells. 72 hours after transient transfection with AS2-Neo vector (Ctl) or AS2-Neo-miR-133a-expressing plasmids, the cells (1 × 10^4^) were seeded in 6-well plates, and the cell numbers were counted on the indicated days. Each conditions were performed in triplicate.


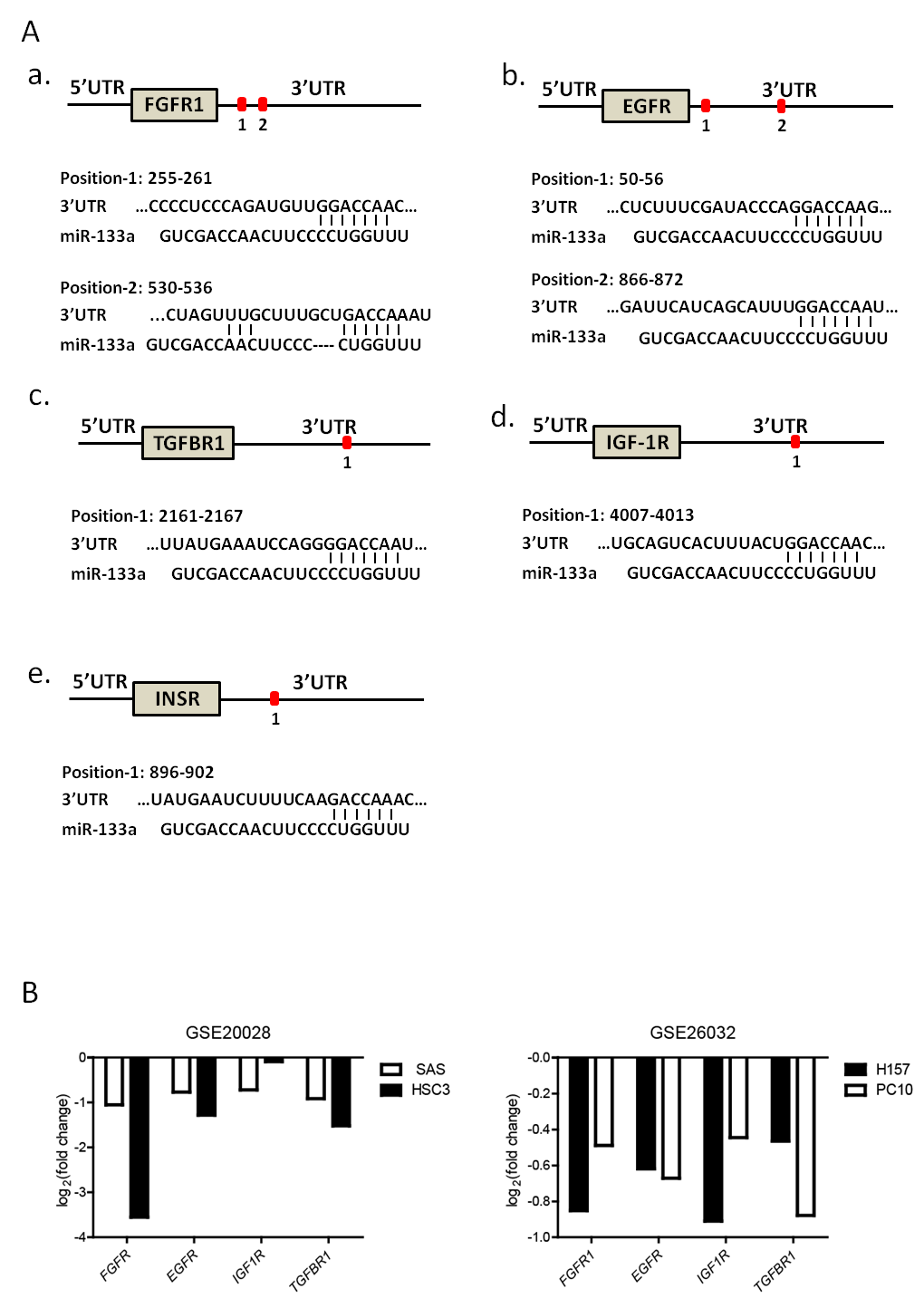


Figure S2. FGFR, EGFR, IGF-1R and TGFBR1 are direct targets of miR-133a.

(A) Putative miR-133a binding sites of the five receptors, including FGFR, EGFR, IGF-1R, INSR and TGFBR1, were identified by computational algorithms from Targetscan (a-e). (B) Fold changes of FGFR, EGFR, IGF-1R and TGFBR1 were estimated from expression profiles (Data set: GSE20028 and GSE26032) of miR-133a-transfected cancer cells vs. control cells. SAS and HSC3 were the oral squamous cell lines; H157 and PC10 were the lung squamous cell carcinoma cell lines.


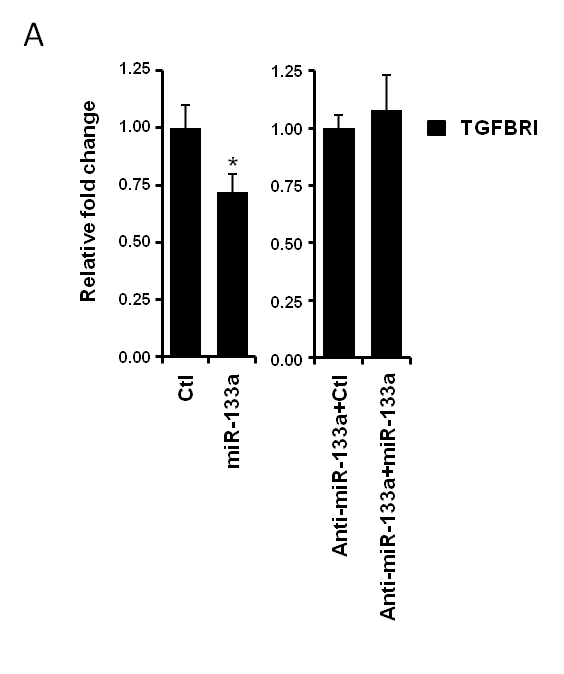


Figure S3. TGFBR1 3’UTR is suppressed by miR-133a.

(A) Co-transfection of CL1-5 cells with AS2-Neo vector (Ctl) or AS2-Neo-miR-133a-expressing plasmid with firefly luciferase fused with 3’UTR of TGFBR1 for 6 hours and then incubated with anti-miR-Ctl or an anti-miR-133a inhibitor (100 nM) in complete medium for 36 hours. Luciferase activity was measured, and the relative ratio of the activity in the miR-133a groups to that in the control vector group is presented.


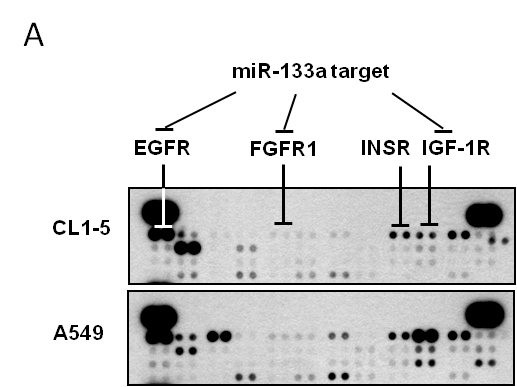


Figure S4. Phospho-receptor detection in CL1-5 and A549 cell lines.

(A) CL1-5 and A549 cell lysates were incubated with nitrocellulose membranes that were conjugated with phospho-receptor antibodies in duplicate. The phosphorylation levels of EGFR, FGFR, INSR and IGF-1R were determined.


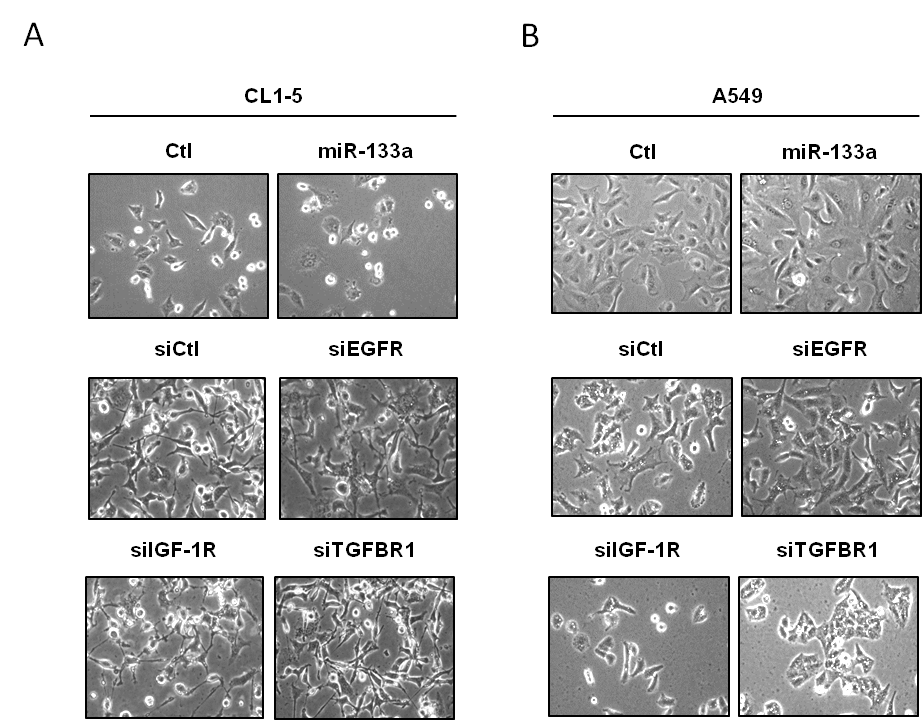


Figure S5. Cell morphology of miR-133a-overexpressing or oncogenic receptor-silenced CL1-5 or A549 cells.

The cell morphology of CL1-5 (A) or A549 (B) was determined 72 hours after transient transfection with AS2-Neo vector (Ctl) or AS2-Neo-miR-133a-expressing plasmids (upper panel) or 48 hours after transient transfection with siCtl, siEGFR, siIGF-1R or siTGFBR1 treatment (middle and bottom panel, respectively).


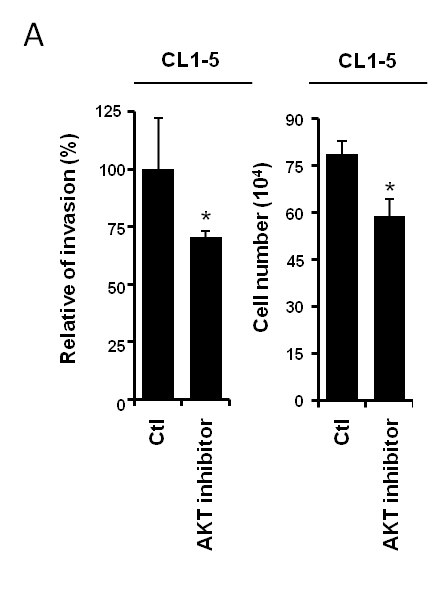


Figure S6. AKT signaling is essential for cell proliferation and cell invasion in CL1-5 cell lines.

(A) CL1-5 cells were pre-treated with the AKT inhibitor (7.5 μM) for 24 hours, and then seeded into chambers with AKT-inhibitor containing medium. The invasive cells were determined 20 hours post-incubation. (B) The cell numbers of CL1-5 were counted after AKT inhibitor treatment for 3 days.


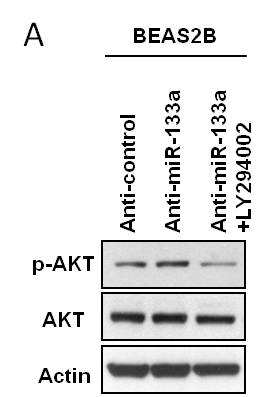


Figure S7. Up-regulation of phospho-AKT mediated by an anti-miR-133a inhibitor can be reduced by the PI3K/AKT inhibitor in BEAS-2B cells.

(A) Representative immunoblots showing the protein levels of pAKT (Ser473), AKT and β-actin in BEAS2B cells after treatment with an anti-miR-133a inhibitor (100 nM) with either DMSO or LY294002 (50 μM) for 48 hours.

**Table S1. miR-133a expression in relation to clinical parameters and pathological characteristics**

|  |  |  | **miR-133a** | | |  |
| --- | --- | --- | --- | --- | --- | --- |
| **Category** | **Subcategory** | **All** | **Low (%)** | | **High (%)** | **P** |
| **Age mean(SD)** |  | **65.88 (12.17)** | **65.82(9.40)** | | **65.92(13.74)** | **0.963^a^** |
| **Cell type** | ***Adenocarcinoma*** | **55 (49.11)** | **23 (52.27)** | | **32 (47.06)** | **0.478^b^** |
|  | ***Large cell carcinoma*** | **7 (6.25)** | **4 (9.09)** | | **3 (4.41)** |  |
|  | ***Squamous cell carcinoma*** | **50 (44.64)** | **17 (38.64)** | | **33 (48.53)** |  |
| **Gender** | ***Male*** | **88 (78.57)** | **35 (79.55)** | | **53 (77.94)** | **1.00^b^** |
|  | ***Female*** | **24 (21.43)** | **9 (20.45)** | | **15 (22.06)** |  |
| **Tumor stage** | **Stage *I*** | **47 (41.96)** | **17 (38.64)** | | **30 (44.12)** | **0.804^b^** |
|  | **Stage *II*** | **28 (25.00)** | **11 (25.00)** | **17 (25.00)** | |  |
|  | **Stage *III*** | **37 (33.04)** | **16 (36.36)** | **21 (30.88)** | |  |

^a^ p-value used t-test to calculate, ^b^ p-value used fisher’s exact test to calculate.
